# Understanding how social norms influence access to and utilization of adolescent sexual and reproductive health services in Northern Nigeria

**DOI:** 10.3389/fsoc.2023.865499

**Published:** 2023-10-11

**Authors:** Modupe Oladunni Taiwo, Oluwatoyin Oyekenu, Rahinatu Hussaini

**Affiliations:** ^1^Save the Children International, London, United Kingdom; ^2^Global Alliance for Improved Nutrition, Abuja, Nigeria

**Keywords:** sexual and reproductive health, child early and forced marriage, social norms exploration, gender transformation, Northern Nigeria

## Abstract

**Background:**

This study explored the influence of social norms on the access and utilization of sexual and reproductive health services by adolescents. Apart from individual and environmental barriers, social norms influence contraceptive decisions and ultimately sexual and reproductive health outcomes. Social norms that shape group behavior describe acceptable standards of behavior and evoke sanctions when such behavior standards are not adhered to. Sexually active adolescents in Nigeria have a relatively low level of modern contraceptive use being influenced by social norms. Scaling up adolescent reproductive health interventions that integrate normative change for a wider impact of programs remains challenging.

**Methods:**

Using data from 18 communities, 188 married and unmarried adolescents (F52% and M48%) and 69 (F37%; M63%) reference group participants were purposively sampled and participated in a social norms exploration intervention study conducted through focus group discussion and in-depth interviews between October and November 2019. The Advancing Learning and Innovation on Gender Norms (ALIGN) Social Norms Exploration Tool (SNET) was adapted for the data collection into discussion guides and vignettes. Pilot testing of the tools informed review and validation prior to actual data collection.

**Findings:**

Low contraceptive uptake by adolescents was characterized by early and forced marriage in childhood; a prominent practice enshrined in social norms around girl-childchastity, family honor, and disapproval of pre-marital sex and pregnancy out of wedlock.

**Conclusion:**

The understanding of harmful social norms, normative change actors, and potential norm-shifting factors for contraceptive decisions by adolescents is essential for effective adolescent sexual and reproductive health interventions for wider impact and adaptive programming in behavior change interventions for improving the access to and utilization of modern contraceptives by adolescents for improved sexual health outcomes, the attainment of the Family Planning (FP) 2030 commitment and universal health coverage policy.

## Introduction

Adolescents and young people aged 10–24 make up one-quarter of the world’s population (UNFPA, 2014). This demography contributes significantly to poor sexual and reproductive health outcomes.Low contraceptive uptake among adolescents in sub-Saharan Africa is a major driver of early pregnancy and abortion as well as increased risk of HIV infection and maternal and child morbidity and mortality (Harrington et al., 2021). Nguyen et al. (2019) noted that early marriage, a reality for millions, restricts educational and vocational opportunities, leading to an intergenerational cycle of poverty.

Still in the formative phase of developing agency, the abilities, capacities, attitudes, and behaviors of young people are determined by the social systems that shape decisions and norms that dictate what is acceptable within the social group (Mackie et al., 2015). Social norms that shape behaviors and actions within a social network have been identified as an important influencer of contraceptive decisions and uptakeby adolescents. In Nigeria, contraceptive prevalence among adolescents 15–19 years is 2%, leaving a large population of adolescents with unmet needs and with its attendant consequences (National Population Commission (NPC) and ICF, 2019).

“Disapproval” by influencers on social networks, which is closely linked with social norms, is perceived as a key factor influencing low contraceptive uptake. However, attempts at describing the influence of social norms on contraceptive use have focused largely on theoretical explanations with limited contextual relevance to the realities of adolescents. Social norms are described as unwritten, obligatory rules that prescribes appropriate and acceptable behaviors in a community and is enforced by the reference group (Stoebenau et al., 2019; Cislaghi and Heise, 2020). A large qualitative U.S. student study identified a series of varied norms that stretch or restrict agency in negotiating sex among adolescents, validating the role of social norms in moderating contraceptive use (Mollborn, 2010; Mollborn, 2016).

With the understanding that social norms are powerful unwritten rules that moderate adolescent contraceptive use, scholars and practitioners in Africa are integrating social norms strategies to address sexual and reproductive health behaviors among adolescents (Cislaghi and Heise, 2020). Howbeit, few studies exist in developing nations on the influence of social norms on contraceptive uptake among adolescents (Ajilore, 2015; Ezenwaka et al., 2020; Mbachu et al., 2021; Agu et al., 2022; Agha et al., n.d.). Married adolescents most especially face many barriers to accessing contraceptives including sociocultural and religious inhibition and patriarchal influence mandating spousal permission to access health care.

The influence of social norms also promotes gender inequalities. Adolescent girls are disproportionally affected by gendered social norms (Ghimire and Samuels, 2014; Vaitla et al., 2017; Ninsiima et al., 2018; Bingenheimer, 2019; Malhotra et al., 2019) in decisions about sexual health services. Social norms approaches have been used in public health-related interventions and are gradually gaining popularity in Nigeria. Of 64.3 million adolescents and young people, over 19% of female adolescents have begun childbearing in Nigeria (National Population Commission (NPC) and ICF, 2019). Sexual and reproductive health information and services for adolescents in Nigeria are shrouded in myths and misconceptions (Taiwo et al., 2019). This has led to unprotected sexual practices with severely poor health outcomes: 11% of girls are married by the age of 15 years, 40% have had vesico-virginal fistular, and the contraceptive prevalence rate is 2% with high unmet needs. Sexual and gender-based violence is experienced by 30% of married adolescents, the infant mortality rate is 132/100,000 live births, maternal mortality is 512/100,000 live births, and 37% of children under 5 years experience malnutrition (National Population Commission (NPC) and ICF, 2019). Power imbalances, gender inequity, and discrimination against women and girls increase their vulnerability to gender-based violence, including denial of access to reproductive health services.

In recent times much interest has been developed in exploring the influence of social norms on sexual and reproductive health behavior and access to contraceptive services (Cislaghi and Heise, 2018; Save the Children, 2020). Some social norms are restrictive and hinder adolescents from making independent decisions about accessing reproductive health services and contraceptives as desired (De Meyer et al., 2014). This problem becomes aggravated with early sexual intercourse (Nalukwago et al., 2018), forced early marriage (Steinhaus et al., 2019), unwanted pregnancy (Smith et al., 2016), and sexually transmitted infections (Scholly et al., 2005). These poor reproductive health outcomes should be averted if adolescent girls make their own decisions about the use of contraception (Svanemyr, 2020).

Evidence from many African countries shows that adolescent girls are forced into early marriage to preserve their virginity and out of marriage birth due to disapproval of contraceptive use (Government of Uganda, United Nations Children’s Fund, 2015; Berhane et al., 2019). These studies concluded that social norms are adopted and internalized at a very young age and strongly influence how adolescents manage their reproductive health choices.This study aims to understand the influence of social norms on the access and utilization of sexual and reproductive health services by perceived adolescents and to identify relevant reference groups who influence and enforce these social norms to inform adaptive programming.

## Materials and methodology

### Study design

For this qualitative research, we selected the focus group discussion (FGD) and in-depth interview (IDI) methods to provide a data source for triangulation, maximizing the benefit of FGD for examining questions within the context social interaction by adolescents and IDI for gathering data on sensitive topics from the social networks. We conducted FDG with 188 married and unmarried adolescents (F52% and M48%) age 10–19 and IDI with 69 (F37% and M63%) reference group participants aged 25 and above between October and November 2019 in Northern Nigeria. The region has the lowest contraceptive uptake among adolescents in Nigeria.

### Study setting

Study activities took place in 18 communities selected from 3 Northern states Gombe, Katsina and Zamfara. Selected communities are clarified as peri-urban and primarily rural with trading and farming as the major occupations. Hausa is the main ethnic group and language with a few minority Fulani. All authors have had long-term research engagement in women’s reproductive health studies and extensive collective experience in social development interventions, working with the adolescent population in the region.

## Sampling and eligibility

Prior to initiating the recruitment of eligible study participants, a series of consultation meetings were held with community leaders, parents of adolescents, husbands/partners for the married adolescents, and other community membersto optimize communication about the study with prospective respondents. Same sex data collectors were paired with participants to secure confidentiality in the discussion of sensitive topics. The team consisted of 16 qualitative interviewers (eight females and eight males) with a background in social science, public health and Monitoring and Evaluation (ME). The principal investigators provided 3 days of training to the field team emphasizing ethical conduct of sensitive research in human subject design especially among adolescents. The FGD and IDI semi-structured interview guides were piloted, revised, and collaboratively translated into Hausa and back into English to optimize comprehension and allow for accurate documenting of responses. The field team worked with health workers/health volunteers and community mobilizers to recruit participants for FGD and IDI from various community venues- homes, community leaders palaces, town halls and markets.

Purposive and snowballing strategies were used for the recruitment of the study participants. The FGD participants were disaggregated by age (10–14, 15–19), sex (female, male), life stage (married and unmarried), and school status (in-school,out of school) to optimize the group dynamics. Adolescents who fell within the demography described above, had parents/guardians willing to provide consent (if <18), and who were at risk of pregnancy were eligible to participate. Adolescents who had ever been sexually active and were currently married or in a sexual relationship were considered “at risk.”The IDI participants were the reference groups, including adult females and males who have an influence on adolescents’ sexual and reproductive health including contraceptive decisions. They were parents, grandparents, and husbands/partners of married or cohabiting adolescents above 25 years and were selected through a purposive and snowball sampling method.

Adolescents were approached in the communities, and eligibility assessed privately by the study staff team. Written informed consent was signed by adolescents 18 and older. Written parental consent and adolescent consent were obtained for participants under 18. The reference groups also provided written consent for participation. Light refreshments were served to participants to compensate for their time. This study was approved by the Federal Ministry of Health-MOH/ADM/621/V.1/299.

## Informed consent and ascent

Written consent was obtained from all participants in both the FGDs and IDI. All participants received compensation for their time and travel and were provided refreshments. Adolescents below 18 years provided parental consent to participate in the study.

### Data collection

The study was conducted in three phases. Phase 1 was the identification and selection of the reference groups using the “my social network” tool. Phase 2 was the identification and conduct of FGD with eligible adolescents and phase 3 was the conduct of IDI with the reference groups.

The adolescent participants were separated into the focus discussion groups as follows:

UVYAF-Unmarried Very Young Adolescents Females (10–14).
UVYAM-Unmarried Very Young Adolescents Males (10–14).
MVYAF-Married Very Young Adolescents Females (10–14).
UOAF-Unmarried Older Adolescents Females (15–19).
UOAM-Unmarried Older Adolescents Males (15–19).
MOAF-Married Older Adolescents Females (15–19).
MOAM-Married Older Adolescents Males (15–19).
HOA-Husbands of Adolescent (no age limit).

The focus group discussions were held at various locations preferred by the adolescents including the health facility hall, the palace of the community chief, mosques, or community town halls with the assurance of safety and security. The IDIs were conducted mostly in private homes to assure confidentiality and safety.

The study adapted the Advancing Learning and Innovation on Gender Norms (ALIGN) social norms exploration phases and tools (developed by the Institute for Reproductive Health at Georgetown University and FHI 360 (2016) and Institute for Reproductive Health (2019), United States), including a range of participatory learning and action (PLA) techniques as part of the Social Norms Exploration Tool (SNET). The 5 Whys, “My Social Network,” and Vignettes were adapted and developed into the interview guides.

The IDIs and FGDs were conducted in the language preferred by participants. The IDIs and FGDs were digitally audio-recorded and simultaneously transcribed and translated into English by the interviewers themselves; a different team reviewed each transcript for accuracy of transcription and translation. Each participant completed a brief tablet-based socio-demographic questionnaire, which was administered verbally by the study staff.

## Data analysis

We used an inductive, thematic approach to analyze the qualitative data.The Co-PIs agreed on a set of initial themes designed to reflect *a priori* domains of interest from the interview guides and exposure to the raw transcripts and field notes. The lead author, who has over 20 years of experience working in Nigeria with expertise in qualitative data analysis, and the co-authors defined the themes and meanings in parallel. After comparing the codes and themes application in two rounds, new themes were added, and the final codebook for the FGDs and IDIs was constructed. Investigators held several meetings to discuss coding discrepancies and emerging themes.The analytic team identified the most significant codes, grouped similar and contrasting excerpts within and between codes, and wrote analytic summaries for each major theme.The analysis identified common themes, recurrent patterns, and essential and interesting or dissenting views.

## Results

Our findings synthesized the social factors at play as adolescents navigate decisions around contraceptive use. We present the primary themes emerging from the analysis: adolescent fertility and desire, an expectation of the achievement of a girl’s purity and family honor through early marriage, the influence of one’s significant other in contraceptive decisions, and social influences on fertility decisions and contraceptive use (Table 1).

**Table 1:** Participant characteristics.

Characteristics	
Adolescents total (*n* = 188)	
*Sex*	
Female	97 (52%)
Male	91 (48%)
*Location*	
Gombe	60 (32%)
Katsina	64 (34%)
Zamfara	64 (34%)
*Marital status*	
Married females 10–14	15 (15%)
Married females 15–19	35 (36%)
Married males 10–14	0 (0%)
Married males 15–19	25 (27%)
Education status(female)	
in school- primary	24 (25%)
in school-secondary	15 (15%)
Out of school	58 (60%)
Reference group (*n* = 69)	
*Sex*	
Female	25 (37%)
Male	44 (63%)
*Location*	
Gombe	26 (38%)
Katsina	27 (39%)
Zamfara	16 (23%)

### Adolescent fertility preference and desire influenced by societal expectations

Understanding adolescent fertility preferences and desires is vital for gaining insight into their contraceptive decision-making. Both married and unmarried adolescents generally placed a high value on pregnancy and childbearing, a perception influenced by social expectations of all girls and women whether currently married or planning to marry in the future. Majority of the respondents consider being pregnant and having many children as a blessing and status conferment that guarantees marriage stability for the wife.

*“It is good to have many children when you are married, this shows that you have a blessing from God” –* Zainab, married adolescent girl, 15 years old, Gombe.

Although pregnancy outside of the marriage is frowned upon as dishonor to the family, pregnancy is celebrated in a marriage even when the wife is an adolescent. The majority of girls resonated less with the concept of “planning” a pregnancy and more with the idea that pregnancy is a “blessing” from God and should be embraced as often as it occurs.

*“As a married person, only God can give you children and you should not reject it when you receive many from him. Children are a blessing in the home”* – Madinatu, married adolescent girl, 17 years old, Zamfara.

This is a notion developed through the enforcement of social expectations that children accompany marriage and where none exist, this becomes a cause for anxiety and non-acceptance in certain networks. Married women without children are described as “barren” and stigmatized.

There is still a patriarchal value ascribed to and preference for male children in key decisions around marriage, pregnancy, and health-seeking practices. Northern Nigeria presents a skewed bias for males in family life in all communities.

*“Many families are happy to have sons in their homes and when all your children are only girls, you are not yet a fruitful wife until you have a son for your husband. Sometimes the husband will marry another wife to have a son for him”* – Mallama Bintu, 69 years old, Katsina.

Very few young unmarried adolescents were ambivalent about getting pregnant and having children with the feeling that a child is valuable and desired in all sexual relationships. Many expressed a desire to be married after completion of primary education.

*“If I get pregnant now, I would just take it easy, I would be happy because pregnancy is not a disease, I only need to carry it for 9 months and deliver and I would have shown that I am fertile.”* Ramatu, unmarried adolescent, 15 years old, Gombe.

### Social influences on fertility decisions and contraceptive uptake

An important social norm hindering the use of contraception is the expectation that having many children guarantees marital security and enhances the social status of women and respect for men in the community. By implication, this has made many women put their health at great risk to fulfill this obligation and be accepted in the social network. Similarly, the social expectation for son preference for family lineage perpetuity poses the burden of having many children on the married couple. Respondents confirmed that this could only be achieved by having many children to keep the family name alive.

*“In our community, a man can only be respected as a man only when he has many children in his house. Also, our religion allows a man to have at least four wives so that he can have many children as a sign of fruitfulness”* Mallam Isha, 64 years old, Zamfara.

The communities do not want to deviate from having large families despite current economic realities and therefore, enforce the norms to prevent uptake of contraception. The value of large family size is the availability of cheap labor on the farm and the continuity of the family business, especially cattle rearing by the sons. Many married adolescents confirmed their husbands’ unwillingness to grant permission to access contraceptives in order to prevent a potential drop in their social rating.

*“Any woman that goes against her husband to get contraceptives services will be seen as feeling bigger than her husband and stands to be punished*” – Amina, married adolescent girl, 14 years old, Gombe.

Many married adolescents feel ostracized from peers for fear of being influenced to use contraceptives without their husbands’ permission.

“*They (the husbands) will not allow her to speak to other women because they can influence her negatively against her husband*”- Abibatu, unmarried adolescent girl, 13 years old, Gombe.

In addition to the shared social expectations by the adolescents, the reference groups of married adolescent females hold powerful, deep-rooted *myths and misconceptions* about the use of modern contraceptives, such as the belief that the use of modern contraceptives causes cancer and damage to the womb, eventually causing infertility. This can be linked to the desire to have many children.

Very poor knowledge about family planning and child spacing was observed among the female reference groups owing to generational denial of healthcare information and services access. In some instances, side effects experienced from off-the-counter contraceptives without appropriate information and counseling created fear of continuing and discouragement of potential users.

### Influence of significant other on contraceptive decision

All household decisions including accessing health care services and contraceptives are deferred to the husband or the mother-in-law. An adolescent wife is considered to be too young to make her own decisions and thus requires protection from the older members of the family. Health facilities are not allowed to offer contraceptives without the permission of the husband.

A movement restriction order is placed on an adolescent wife when no family member is available to be in her company. In the traditional African context and many contexts, mothers-in-law are highly esteemed by the wives and play significant roles in disapproval of contraceptive use by the wives.

“*A woman cannot go out of the house alone, someone must accompany her to her destination even if she wants to visit her mother. Many mothers-in-law do not want us to use contraceptives so as not to prevent pregnancy. We cannot disrespect our mother-in-law, we just agree with them and move on. Their role is to take care of our children and they are always happy when a new baby is born.”* Mariatu, married adolescent girl, 19 years old, Katsina.

### Achievement of girl’s purity and family honor through early and forced marriage

Early and forced marriage is a common practice across the three states but mainly in the rural areas as a means to enforce girls’ purity and family honor. Where child marriage happens, the young girls are socialized to believe it is normal and have accepted it. As stated by the participants, the average age of marriage was between 12 and 15 years. Traditionally, girls at the age of 15 years are regarded as suitable and ready for marriage. Early marriage signifies early childbearing and the opportunity to have many children before getting old. Most marriages are contracted with older men who make the overall decisions, including contraceptive use.

*“Many young girls in this community marry early because our mothers and grandmothers too married early. We are happy to be married because we get a lot of nice clothes and shoes from our husbands. Our mothers tell us that our husbands will take good care of us if we continue to have many children”-* Adijatu, married adolescent girl, 16 years old, Katsina.

The prominent social norm influencing the practice of child marriage is the expectation of chastity and virginity preservation for girls to promote family honor and dignity. Pregnancy by girls outside of marriage is considered shameful.

The pregnant girl also risks being disowned, and the baby carries the label of illegitimate with a life-long stigma. Potential suitors would most likely avoid marriage to other female siblings from that family and be marked as “not well brought up.” Adolescent girls are socialized from a very young age to carry the burden of preserving the family name by getting married early and having many children.

*“When a girl is 15, she is regarded as an adult, which is the height of her beauty (that is when she can get the best suitors). Men that follow her start reducing afterwards. Furthermore, if she gets to the age of 20, she gets boldness to talk back at her parents, and she can even become promiscuous because she would not listen to her parent”* Mariam, unmarried adolescent girl, 14 years old, Katsina.

Interestingly, while a few mothers interviewed in the reference group were not happy with this tradition and the practice of early marriage, the fear of losing the highly regarded family honor when their daughter becomes pregnant out of wedlock and being mocked by society makes them conform. Mothers desire education for their young girls up to at least senior secondary school, but the pressure and fear put on them by society makes them submit to marrying the girls off by 15 years old.

*“It is not all the time that I am happy to marry my girls out, anytime the man comes to our husband, I feel very sad that another girl in my household is going to marry a man she does not know very well, but I do not have a say in the marriage decision. Only my husband and his brothers and his uncles plan the wedding”*- Mallama Sadia, 42 years old, Zamfara.

Adolescent males also expressed the view that some parents fear not meeting social expectations of marrying their daughter off by the time she attains puberty. This places a lot of pressure on the girl’s parents and may lead to mockery from the community. Because of this fear, the parents give out their daughters in marriage to whoever shows interest to find acceptance in their communities.

*“When my sister was being prepared for wedding at the age of 13, I was not happy because I will be going to school alone. She was in class 4 then and my father gave her out to a man from Dadin Jaji village. Now my sister is 20 and has 4 children. She is not using any contraceptive because her husband wants seven children”*- Abubakar,19 years old, Zamfara.

Similarly, most unmarried older adolescent girls believe that puberty is a signal for marriage to safeguard against being promiscuous and dishonoring the family name.

*“Once a girl reaches the age of puberty, she should be married off because puberty leads to sexual desire; hence parents should give the girls out in marriage to prevent promiscuity and use of contraceptive that will destroy her womb.**”*** Jumai, unmarried adolescent 19 years old, Gombe.

Other social factors were attributed to child marriage and denial of contraceptive use, including fear of not finding a suitable suitor when they get too old.

## Discussion

From the 186 adolescents in the FGD and 69 reference group participants who participated in this community-based study, we elicited a strong preference for getting pregnant and having many children as a sign of maintaining societal status and fulfilling expectations. Contraceptive decisions by adolescents were significantly influenced by social norms enforcing the practice of early marriage, high fertility, and son preference. Early and forced marriage was a crucial driver of low contraceptive use. This agrees with Steinhaus et al. (2019), who reported that child marriage in Uganda, influenced by the norm of maintaining family honor, hinders contraceptive use in adolescent girls. The significant social norm promoting child marriage that “girls are expected to maintain chastity and not get pregnant before marriage” is aligned with a finding shared by Smith et al. (2016). When this happens, a girl is deemed to have brought shame to her family, stands the chance of being disowned by her parents, and her child stigmatized. In addition, a girl is expected to preserve the name and honor of their family until marriage. It is believed that when a girl gets pregnant out of wedlock, this brings shame to their family, who also face backlash and may be ostracized by the wider community.

The decision to have the girls marry early supports high fertility and increases the risk of poor sexual health outcomes. The concept that power can be demonstrated in decisions to “adhere to (or not adhere to) social norms” in Pulerwitz et al. (2029) is relevant to our analysis. Contraceptive use by adolescents is generally viewed as a non-conformity to social norms, which impacts independent sexual and reproductive health decisions and contraceptive use. The adolescents in the study communities have lost their agency and power to seek health services when required without spousal permission.

In order to address the influence of social norms on contraceptive decision-making by adolescents, intervention to stimulate behavior change toward contraceptive uptake should target individuals and communities and focus on supporting adolescent decision-making agency and empowerment to challenge the inhibiting norms. In addition, an enabling social environment should be fostered through engagement with key influencers to promote behavior transformation toward acceptance of contraceptive use by adolescents. Provider initiated contraceptive outreach interventions should integrate strategies for addressing pervasive social messaging associating contraceptive use with infertility. Husbands of adolescents and mothers-in-law are the main referents in continuing gender-discriminatory behaviors in adolescent reproductive and sexual health. Similarly, mothers-in-law and grandmothers are the key influencers of disapproval of contraceptive use and advocates for having many children (Dixit et al., 2022). It is therefore critical to channel efforts at shifting harmful social and gender norms to target men/husbands, mothers, grandmothers, and mothers-in-law.

## Conclusion

Using the social norms exploration tools to deepen our understanding of the norms influencing adolescent sexual and reproductive health, especially contraceptive use and other interconnected behaviors including, fertility preference and desire, child marriage, and influence of significant others in contraceptive decision-making in Northern Nigeria, there is evidence of intersectionality among the behaviors. Fertility decisions of adolescents are largely influenced by societal expectations and conformity to the desires of husbands and mothers-in-law without consideration for adolescent preference. Early and forced marriage is a prominent practice that supports high fertility and disapproval of contraceptive use. Unequal power relations, low uptake of modern contraceptives with implications for poor maternal and child health outcomes, increased risk of sexually transmitted infections, maternal and infant morbidity and mortality, and overall poor well-being for women and girls are the result of the enforcement of harmful social norms. With an improved understanding of the social norms influencing the utilization of sexual and reproductive health services and the key influencers, professionals can develop appropriate responses to address the impact of these social norms. This knowledge allows for direct engagement with the key influencers through a human-centered intervention that empowers influencers to self-reflect and develop context-relevant solutions and actions to promote positive social norms within the social network.

## Data availability statement

The raw data supporting the conclusions of this article will be made available by the authors, without undue reservation.

## Ethics statement

The studies involving humans were approved by name and affiliation of the ethics committee/institutional review board MOH/ADM/621/V.1/299. The studies were conducted in accordance with the local legislation and institutional requirements. Written informed consent for participation in this study was provided by the participants’ legal guardians/next of kin.

## Author contributions

MT was the principal investigator, developed the research concept, and provided leadership in the research development, data collection, analysis, and report. OO supervised the research study and contributed to the development of the manuscript. RH contributed to the design of the study tools and pre-testing. All authors contributed to the article and approved the submitted version.
